# What is the impact of metabolic dysfunction-associated steatotic liver disease (MASLD) on outcomes in people living with chronic hepatitis B (CHB)? An umbrella review protocol

**DOI:** 10.12688/wellcomeopenres.26466.1

**Published:** 2026-05-14

**Authors:** Emily Martyn, Jessica Carter, Maeve Barlow, Flora Olcott, Indrajit Ghosh, Elizabeth Waddilove, Alejandro Arenas-Pinto, Richard Gilson, Stuart Flanagan, WD Francois Venter, Jennifer Manne-Goehler, Sally Hargreaves, Emmanouil Tsochatizis, Douglas MacDonald, Philippa C Matthews

**Affiliations:** 1 The Francis Crick Institute, London, England, UK; 2 Division of Infection, Immunity and Transplantation, University College London Faculty of Medical Sciences, London, England, UK; 3 Migrant Health Research Group, Institute for Infection and Immunity, City St George's, University of London, London, UK; 4 Wolfson Institute of Public Health, Queen Mary University of London - Charterhouse Square Campus, London, England, UK; 5 Mortimer Market Centre, Central and North West London NHS Foundation Trust, London, England, UK; 6 Institute for Global Health, University College London Faculty of Population Health Sciences, London, England, UK; 7 Find & Treat, University College London Hospitals NHS Foundation Trust, London, England, UK; 8 Ezintsha, University of the Witwatersrand Johannesburg Faculty of Health Sciences, Johannesburg, Gauteng, South Africa; 9 Division of Infections Diseases, Brigham and Women's Hospital Department of Medicine, Boston, Massachusetts, USA; 10 Medical Practice Evaluation Center, Massachusetts General Hospital, Harvard Medical School, Boston, Massachusetts, USA; 11 Royal Free Hospital Sheila Sherlock Liver Centre, London, England, UK; 12 UCL Institute for Liver and Digestive Health, London, England, UK; 13 Division of Infection, University College London Hospital NHS Foundation Trust, London, UK

**Keywords:** Chronic hepatitis B, Hepatitis B virus, viral hepatitis, metabolic dysfunction-associated steatotic liver disease, non-alcoholic fatty liver disease, metabolic dysfunction-associated fatty liver disease, MASLD, MAFLD, NAFLD, fatty liver, umbrella review

## Abstract

**Introduction:**

An estimated 254 million people are living with chronic hepatitis B virus (CHB) and 1.6 billion with metabolic dysfunction-associated steatotic liver disease (MASLD) worldwide. CHB remains a major global health challenge, despite a robust vaccine and suppressive antiviral treatment, and MASLD prevalence is increasing in line with a rise in cardiometabolic risk factors, such as obesity and type 2 diabetes. Thus, understanding the impact of concurrent CHB and MASLD on liver health is important to inform clinical practice and population surveillance. Current literature, including over ten systematic reviews and meta-analyses, yields conflicting evidence about the interplay between CHB and MASLD. This protocol describes a study aiming to critically appraise published systematic reviews investigating the effect of MASLD on liver outcomes in people living with CHB, identify gaps in the available data and assist scientists and clinicians in informing future research design.

**Methods and analysis:**

We will use umbrella review methodology from the Joanna Briggs Institute (JBI) to evaluate and synthesise evidence from existing systematic reviews and meta-analysis investigating liver and viral outcomes (fibrosis, cirrhosis, hepatocellular carcinoma, response to antiviral treatment, and impact on CHB biomarkers e.g. hepatitis B surface antigen, hepatitis B e-antigen and viral load) in CHB and MASLD, compared to CHB alone. We will search Ovid Medline, Ovid Embase, PubMed and Web of Science. We will assess methodological quality using the Assessing the Quality of Systematic Reviews-2 (AMSTAR-2) tool, compare study characteristics and present important results.

**Ethics and dissemination:**

No ethics approval is required as we will use only data from systematic reviews that are already published. Results will be included in a PhD thesis, submitted for consideration for presentation at international conferences and for peer review for publication in a scientific journal. We will endeavour to share results with relevant public and patient organisations.

**Registration details** This umbrella review has been published on PROSPERO (ID CRD420261308282).

## Plain Language summary

Around the world, an estimated 254 million people are living with chronic hepatitis B (CHB, a long-term viral liver infection) and about 1.6 billion people have metabolic dysfunction-associated steatotic liver disease (MASLD), which as a condition caused by too much fat in the liver, commonly associated with obesity, diabetes, high blood fats and high blood pressure. Both CHB and MASLD are major causes of liver disease, and many people have both at the same time. Understanding how these two diseases interact is important for improving patient care and public health planning.

Systematic reviews and meta-analyses are a way of collecting and summarising all existing research, and combining data to produce an overall estimate to answer a question. There have been several systematic reviews and meta-analyses investigating the effect of having both CHB and MASLD on patient outcomes, including liver scarring (fibrosis), advanced liver disease (cirrhosis), liver cancer, response to antiviral treatment, and key markers of CHB in the blood, but the results are inconsistent. Some studies suggest MASLD worsens liver disease in people living with CHB, while others do not. This makes it difficult to draw conclusions on best patient care.

This study will look very carefully at the pubished systematic reviews and meta-analyses in this field, by conducting an “umbrella review”. We will search major medical databases to find relevant studies, then assess the quality of each review using a recognised tool to ensure reliability. We will compare findings across studies, highlight key results and suggest reasons for differences.

By summarising the best available evidence and identifying gaps in current knowledge, this study aims to support clinicians and researchers in better understanding the combined impact of MASLD and CHB, and to guide future research in this area.

**Abbreviations**
ALT: Alanine Transaminase
AMSTAR-2: A Measurement Tool to Assess systematic Reviews-2
BMI: Body Mass Index
CHB: Chronic Hepatitis B
CLD: Chronic Liver Disease
HBeAg: Hepatitis B e-Antigen
HBsAg: Hepatitis B Surface Antigen
HBV: Hepatitis B Virus
HCC: Hepatocellular Carcinoma
HDL: High Density Lipoprotein
JBI: Joanna Briggs Institute
MAFLD: Metabolic Dysfunction-Associated Fatty Liver Disease
MASLD: Metabolic Dysfunction-Associated Steatotic Liver Disease
NAFLD: Non-Alcoholic Fatty Liver Disease
SLD: Steatotic Liver Disease
VL: Viral Load
WHO: World Health Organization

## Definitions

**Table:** 

Term	Definition
Steatotic Liver Disease (SLD)	An overarching term used to describe any liver condition leading to abnormal fat accumulation (>5% hepatocytes containing lipid droplets or fat contributing to 5% of liver weight) in the liver (liver, or hepatic, steatosis) ^1^
Metabolic dysfunction- associated steatotic liver disease (MASLD) ^2^	SLD plus at least one cardiometabolic risk factor: • Pre-diabetes or diabetes (Fasting blood glucose ≥5.6 mmol/L or 2 hour post load glucose levels ≥7.8 mmol/l or HbA1c ≥ 39 mmol/mol or diabetes specific treatment), • Body Mass Index >25 kg/m ^2^ (≥ 23 kg/m ^2^ Asian populations) or waist circumference: ○ ≥94 cm in men and ≥ 80 cm in women (Europeans) ○ ≥90 cm in men and ≥ 80 cm in women (South Asians and Chinese) ○ ≥85 cm in men and ≥ 90 cm in women (Japanese) • Blood triglycerides ≥1.7 mmol/l or lipid-lowering treatment, • Blood HDL-cholesterol <1.0 (men) / <1.3 mmol/l (women) or lipid-lowering treatment, • Blood pressure ≥ 130/80 or specific hypertension treatment.
Metabolic dysfunction-associated fatty liver disease (MAFLD) ^3^	SLD plus: • overweight/obesity (BMI ≥ 25 kg/m ^2^ or ≥ 23 kg/m ^2^ in Asian populations), or • type 2 diabetes (according to widely accepted international criteria), or • ≥ 2 of: ○ Waist circumference (≥ 102 cm men, ≥ 88 cm women; Asian population - ≥ 90 cm men, ≥ 80 cm women), ○ Blood pressure ≥ 130/80 or specific hypertension treatment, ○ Blood triglycerides ≥1.7 mmol/l or lipid-lowering treatment, ○ Blood HDL-cholesterol <1.0 (men) / <1.3 mmol/l (women) or lipid-lowering treatment, ○ Pre-diabetes (HbA1c 39–47 mmol/mol or fasting blood glucose 5.6–6.9 mmol/l or 2 hour post load glucose levels 7.8–11 mmol/l) ○ Homeostatic model assessment for insulin resistance (HOMA-IR) ≥ 2.5 ○ C-reactive protein >2 mg/L
Non-alcoholic fatty liver disease (NAFLD)	SLD in individuals who drink little or no alcohol (<20 g/day females, <30 g/day males) and no other cause of steatosis e.g. medications, monogenic disorders. ^4^

## Introduction

Chronic Hepatitis B (CHB) and metabolic dysfunction-associated steatotic liver disease (MASLD) are major global causes of chronic liver disease. ^5^ Despite an effective, robust vaccine and suppressive antiviral treatment, CHB causes an estimated 1.1 million deaths annually. ^6^ MASLD occurs when there is excess fat in the liver (steatosis, defined as >5% of hepatocytes containing fat deposition) together with at least one cardiometabolic risk factor (overweight/obesity, diabetes, hypertension or dyslipidaemia). ^2^ MASLD prevalence is rising in the context of a global increase in obesity and type 2 diabetes, and a critical convergence of MASLD in people living with CHB is emerging. ^7– 9^

The acronym MASLD was introduced in 2023, to move away from potentially pejorative language and to acknowledge cardiometabolic factors in liver disease pathogenesis. Multiple terms exist in the literature, including metabolic dysfunction-associated fatty liver disease (MAFLD) and non-alcoholic fatty liver disease (NAFLD), ^2^ although several analyses suggest that MASLD, MAFLD and NAFLD are highly concordant and have consistent clinical outcomes. ^10^ In the last five years, multiple systematic reviews and meta-analyses have been published on the effect of concurrent MASLD (or NAFLD/MAFLD) in people living with CHB. ^11– 20^ However, the conclusions drawn by these reviews are conflicting, and the underlying reasons for inconsistencies are not fully understood. ^21, 22^ Suggestions include heterogenous study design, diverse populations studied, and differences in SLD definitions.

We are not currently on track to achieve World Health Organization (WHO) viral hepatitis elimination targets, which include a 90% reduction in incidence and 65% reduction in mortality from viral hepatitis by 2030. ^23, 24^ In addition, hepatocellular carcinoma mortality rates continue to rise globally, with MASLD and CHB remaining leading causes. ^25^ As part of an effort to address targets, WHO widened CHB treatment eligibility in revised 2024 guidelines, including MASLD as a potential treatment initiation criterion, despite uncertain evidence. ^26^ Moreover, new pharmacological treatments are becoming available for MASLD, but trials exclude CHB. ^27^ Thus, we urgently need to better understand the available data in order to inform our approach to MASLD in people living with CHB and guide future research and clinical care.

Our objective is to collate, systematically and critically evaluate, and identify gaps in the data for the effect of MASLD on outcomes of HBV infection, including HBsAg clearance (functional cure), response to antiviral therapy, and rates of liver inflammation, fibrosis, cirrhosis and hepatocellular carcinoma in people living with CHB. To address this objective, we here present a protocol for an umbrella review of systematic reviews, using Joanna Briggs Institute methodology. ^28^

### Objectives

Our umbrella review will use published systematic reviews and meta-analyses to:

• Describe and summarise differences between people living with CHB in the presence vs. absence of MASLD, with respect to:
  ○ Hepatitis B viral load (VL) suppression with and without antiviral treatment,
  ○ Rate of hepatitis B surface antigen (HBsAg) seroconversion with and without antiviral treatment,
  ○ Other antiviral treatment response (including biochemical e.g. alanine transaminase (ALT) normalisation, viral e.g. hepatitis B e antigen (HBeAg) seroconversion)
  ○ Liver inflammation (ALT), fibrosis, cirrhosis and hepatocellular carcinoma (HCC).
• Compare and contrast study methodology.
• Assess systematic review study quality.

## Methods and analysis

### Study team

This protocol has been created by a multidisciplinary team of clinicians, scientists with expertise in viral hepatitis and MASLD, and methodologists.

### Inclusion and exclusion criteria

We define inclusion and exclusion criteria based on population, exposure, comparator and outcomes (See Table 1). Definitions are deliberately broad as we aim to capture all published systematic reviews and meta-analyses, and terminology is wide-ranging in this field (potentially contributing to heterogeneity). This will allow us to systematically describe, compare and contrast the available data, rather than performing further statistical analysis which would require us to combine homogeneous data.

**Table 1.** Inclusion and exclusion criteria for a proposed umbrella review seeking to evaluate published systematic reviews investigating the impact of metabolic dysfunction-associated steatotic liver disease (MASLD) on liver and viral outcomes in people living with chronic hepatitis B (CHB).

Category	Inclusion criteria	Exclusion criteria
**Population**	• Adults ≥18 years • CHB	• Children/young people <18 years • Animals • Not living with CHB
**Exposure**	• MASLD • MAFLD • NAFLD • SLD • Hepatic steatosis	• No SLD
**Comparator**	• CHB alone (no SLD)	N/A
**Outcome**	• HBV viral load e.g. mean difference between MASLD and non-MASLD groups (cross sectional studies), change over time (longitudinal studies). • HBsAg seroclearance (loss of detectable surface antigen in the blood) in people living with CHB not on antiviral treatment. • Risk (e.g. hazard, risk or odds ratio), incidence or prevalence of: ○ Liver inflammation (ALT > ULN*) ○ Liver cirrhosis ○ Liver fibrosis ○ Hepatocellular carcinoma • Response to antiviral treatment (NAs and/or interferon) ○ Biochemical (e.g. ALT normalisation) or viral (e.g. HBeAg or HBsAg seroconversion, viral load suppression)	• Reviews not reporting on liver or viral outcomes in CHB and MASLD. • Studies only reporting on diagnostic test characteristics.
**Study characteristics**	• Meta-analysis • Systematic review • Published in any country, since inception of databases until 28th January 2026 • Peer-reviewed	• Individual observational studies • Randomised controlled trials • Narrative reviews • Diagnostic accuracy studies • Letter to the editor • Conference abstracts • Not in English language

Abbreviations: ALT - alanine transaminase, CHB - chronic hepatitis B, HBeAg - hepatitis B e-Antigen, HBsAg - hepatitis B surface antigen, HBV - hepatitis B virus, MAFLD - metabolic dysfunction-associated fatty liver disease, MASLD - metabolic dysfunction-associated steatotic liver disease, NAFLD - non-alcoholic fatty liver disease, NA - nucleot(s) ide analogues, SLD - steatotic liver disease, ULN - upper limit of normal.

CHB describes a spectrum of disease usually characterised by the presence of detectable HBsAg in the blood or serum for longer than 6 months. ^29^ If analyses don’t apply this strict definition, they can still be included but we will note the definition used. Although MASLD is the most up to date terminology, we will also include studies referring to the older terms NAFLD, MAFLD and the over-arching term SLD, and histopathological term hepatic steatosis. ^2, 30^

Due to significant variation in statistical analysis approaches, we will record the measure used by each study, but will not attempt to pool results. Further information on the types of outcomes that will be described are presented in Table 1.

### Search strategy

Our search timeframe will be from database inception until 28th January 2026. We will use Boolean operators to combine searches. We will search Ovid Medline, Ovid Embase, Web of Science and PubMed. These databases were chosen to provide a broad coverage of the medical literature. ^18^ The search strategy for each database is presented in Table 2.

**Table 2.** Table describing search strategies for target databases for an umbrella review seeking to evaluate published systematic reviews investigating the impact of metabolic dysfunction-associated steatotic liver disease (MASLD) on liver and viral outcomes in people living with chronic hepatitis B (CHB).

Database	Content focus	Search strategy
Ovid MEDLINE	Biomedical and clinical medicine.	1. (“systematic review” or meta-analys*).mp 2. (“metabolic dysfunction-associated fatty liver disease” or “metabolic dysfunction-associated steatotic liver disease” or “non-alcoholic fatty liver disease” or “fatty liver” or “hepatic steatosis” or “liver steatosis” or “steatotic liver disease” or NAFLD or MASLD or MAFLD).mp 3. (“hepatitis B” or “chronic hepatitis B” or “hepatitis B virus” or “viral hepatitis” or HBV or CHB).mp
Ovid Embase	Broad biomedical and pharmacological coverage.	
Web of Science	Multidisciplinary: science, social science, arts/humanities.	1. “systematic review” OR meta-analys* (Topic) 2. (“metabolic dysfunction-associated fatty liver disease” OR “metabolic dysfunction-associated steatotic liver disease” OR “non-alcoholic fatty liver disease” OR “fatty liver” OR “hepatic steatosis” OR “liver steatosis” OR “steatotic liver disease” OR NAFLD OR MASLD OR MAFLD) (Topic) 3. “hepatitis B” OR “chronic hepatitis B” OR “hepatitis B virus” OR “viral hepatitis” OR HBV OR CHB (Topic) 4. #3 AND #2 AND #1
Pubmed	Biomedical, life science, PubMed Central free full text and preprints	1. “systematic review” OR meta-analys* [title/abstract] 2. (“metabolic dysfunction-associated fatty liver disease” OR “metabolic dysfunction-associated steatotic liver disease” OR “non-alcoholic fatty liver disease” OR “fatty liver” OR “hepatic steatosis” OR “liver steatosis” OR “steatotic liver disease” OR NAFLD OR MASLD OR MAFLD) [title/abstract] 3. “hepatitis B” OR “chronic hepatitis B” OR “hepatitis B virus” OR “viral hepatitis” OR HBV OR CHB [title/abstract] 4. #3 AND #2 AND #1

Abbreviations: CHB - chronic hepatitis B, HBV - hepatitis B virus, MAFLD - metabolic dysfunction-associated fatty liver disease, MASLD - metabolic dysfunction-associated steatotic liver disease, NAFLD - non-alcoholic fatty liver disease, .mp - “multi-purpose” = title, book title, abstract, original title, name of substance word, subject heading word, floating sub-heading word, keyword heading word, organism supplementary concept word, protocol supplementary concept word, rare disease supplementary concept word, unique identifier, synonyms, population supplementary concept word, anatomy supplementary concept word. Topic = title, abstract, author keywords, Keywords plus, tiab = title, abstract

### Study selection

Search results will be uploaded into Covidence™ systematic review software, ^31^ and citations will be deduplicated by the software. Two members of the study team will independently review each abstract, then the short list of full text articles, for eligibility using the pre-specified inclusion and exclusion criteria. Conflicts will be resolved through discussion between reviewers. If an agreement cannot be reached, a third reviewer will be consulted.

### Data extraction

Data extraction of descriptive study characteristics will be conducted by two independent reviewers for a sample (30%) of the systematic reviews using a data extraction tool, adapted from JBI Umbrella Review Manual, and piloted in advance by two reviewers ( Table 3). ^28^ A pilot version of a data extraction form will be trialed on Covidence™. A back up Microsoft Excel data collection tool will be available for use depending on the result of the pilot. Results will be compared and, if there is good (>90%) agreement, the remainder of the data will be extracted by one reviewer. Critical evaluation of systematic review quality will be conducted independently by two independent reviewers for all included studies. In cases where the two reviewers cannot agree, or are unsure, advice will be sought from a third expert reviewer.

**Table 3.** Data extraction tool for characteristics of systematic reviews and meta-analysis included in a proposed umbrella review to investigate the impact of metabolic dysfunction-associated steatotic liver disease (MASLD) on liver and viral outcomes in people living with chronic hepatitis B (CHB). Not all information will be available for every study. For each box, the reviewer should document if this study is not available. This will be adapted to a Covidence™ data extraction template.

Study information		Data
Study ID		*Number assigned to study for this review (consecutive 1.x)*
First author, year, DOI		*E.g. Wang, 2023*
Last author country		*Based on senior author institution*
Study countries		*If reported in the review, include information on the countries of publication of the studies included.*
Country of origin of participants		*If there is any information of the country of origin or ethnicity of participants (rather than just the country of recruitment) detail this here.*
Study designs included in the review		*Detail if there are any restrictions on the type of study included in the review e.g. longitudinal cohort only.*
Search strategy		*Note if a search strategy was specified in the methods.*
Sources searched		*Which databases do the authors use? E.g. PubMed, Web of Science etc.*
Start/End date		*Detail the search date range included in the review.*
Study funding sources/possible conflict of interests		*Detail any funding sources identified in the review and any possible conflict of interest for study authors.*
Population description		*E.g. Inpatient vs outpatient* *Primary vs secondary care* *Rural vs urban* *There may be more than one setting for each systematic review.* *Document if the review is geographically restricted*
Inclusion criteria		*Record inclusion criteria.*
Exclusion criteria		*Record exclusion criteria.*
Definition of MASLD used in the review		*MASLD/NAFLD/MAFLD/HS/SLD/other (please specify). Document the thresholds used e.g. steatosis was defined as CAP > 248 dB/m.*
Total number and proportion of participants with MASLD		*How many people (number and percentage) meet the study definition of MASLD/MAFLD/NAFLD/HS/SLD?*
Method of liver steatosis diagnosis		• *Imaging/biopsy/other (specify).* • *More than one method may be used.* • *Document the number and proportion of participants diagnosed by each method (if available).* • *Document the number of studies using this method (some studies may use multiple methods)* • *This will be incorporated into a table in the final data extraction form.*
Primary outcomes		*Record the primary outcomes of the systematic review, as defined by the study authors.* *Include the exact definition of the outcome if possible e.g. “biochemical response” - as defined by normalisation of alanine transaminase within 1 year following antiviral treatment*
Secondary outcomes		*Record the secondary outcomes of the systematic review, as defined in the study authors.*
Total number of studies included		*This should be included in the results section. There may be a different number of studies used for different analyses within the same paper. Record how many studies were used for each analysis, if relevant.*
Total number of participants included		*Document the total number of participants included in the analysis here. Note that the number of participants may also vary depending on the analysis and there will be a space to record this in the “results” section of the data extraction form.*
Baseline population characteristics		*Record the following baseline characteristics, including for the total population, MASLD only and no MASLD, if available:* • *Age (median IQR)* * • *Male sex (n, %)* • *HBV antiviral treatment (any) (n,%)* • *Interferon (n,%)* • *Nucleos(t) ide analogue (n,%)* • *Hepatitis e antigen status (n,%)* • *Quantitative surface antigen (median, IQR)* * * *if the median, IQR is not described, record the descriptive characteristics available in the systematic review (and document the statistics used)*
Antiviral treatment	Complete virological response	*For each result reported in the review document:* • *Result metric (odds/risk/hazard ratio)* • *Confidence interval* • *P-value * • *Number of studies/participants included in the specific analysis* • *Document the result for each reported time point if longitudinal study* • *This will be formatted into a table for each outcome result in the Covidence™ data extraction template.*
	e-antigen loss/sero-conversion	
	Biochemical response	
	s-antigen loss	
Liver outcomes	HCC risk	
	Fibrosis risk	
	Cirrhosis risk	
Viral load off antiviral treatment		• *These will be free text boxes as there will be variation in the reporting of these results.* • *Describe the metric of measurement e.g. median (HBV viral load in people living with CHB only, compared to CHB and MASLD).* • *Report confidence interval and p-value.* • *Describe follow up period if relevant.*
Surface antigen loss/change off antiviral treatment		
Other outcomes		*Free text to describe any other important outcomes documented in the study, not captured by the data extraction tool.*
Comments		*Please record anything you think may be relevant about the study that wasn’t captured in the above fields e.g. reporting clarity, location of certain data fields in the manuscript. It is not necessary to fill this box.*

Adapted from. ^28^ If data are not available, write “n/a”.

* These metrics may be reported as a number for the total population or as summary statistics for people with and without MASLD. Record both if available. Abbreviations: CAP - controlled attenuation parameter, DOI - digital object identifier, HS - hepatic steatosis, MASLD - metabolic dysfunction-associated steatotic liver disease, MAFLD - metabolic dysfunction-associated fatty liver disease, NAFLD - non-alcoholic fatty liver disease, SLD - steatotic liver disease,

### Analysis of the evidence

We will collate descriptive characteristics of each systematic review and meta-analysis and use descriptive tables and figures to compare and contrast our findings. Data will be downloaded from the Covidence extraction tool for further analysis in R studio.

Critical evaluation of systematic review quality will be achieved using the AMSTAR-2 tool ( **A M**ea **S**urement **T**ool to **A**ssess systematic **R**eviews- **2**), developed specifically for systematic reviews including observational studies. ^32^ This tool can be reviewed online ( https://amstar.ca/Amstar_Checklist.php), and comprises 16 domains with 7 highlighted as “critical” due to their potential impact on the validity of the results, as follows:

• Protocol registered before commencing the review;
• Adequacy of the literature search;
• Justification of excluding individual studies;
• Risk of bias from individual studies being included in the review;
• Appropriateness of meta-analytical methods;
• Consideration of bias when interpreting the review;
• Assessment of presence and likely impact of publication bias.

Two team members will complete the AMSTAR 2 tool for each systematic review, then make a judgement on their confidence (critically low, low, moderate or high) in the review results based on their assessment of methodological quality ^32^ ( Table 4). A Microsoft Excel™ version of this tool will be used to collate the evidence.

**Table 4.** AMSTAR 2 tool confidence rating. ^32^ Sixteen domains will be used to evaluate the quality of the systematic review methodology. An interactive version of the tool can be accessed online ( https://amstar.ca/Amstar_Checklist.php). Seven of the domains are considered critical as they can fundamentally affect the validity of the review. Based on this, an overall “confidence rating” for the review results can be established. This is not intended to be a score, more an overall view from the reviewer on the potential impact of the inadequate domains on the review quality and therefore confidence that should be placed on its results.

Confidence	Description
High	*No or one critical* * *weakness*: “the systematic review provides an accurate and comprehensive summary of the available studies that address the question of interest”.
Moderate	*More than one non-critical weakness*: “the systematic review has more than one weakness but no critical weaknesses. It may provide an accurate summary of the results of the available studies that were included in the review.”
Low	*One critical weakness, with or without non-critical weaknesses:* “the systematic review has a critical flaw and may not provide an accurate and comprehensive summary of the available studies that address the question of interest.”
Critically low	*More than one critical weakness, with or without non-critical weaknesses:* “the systematic review has more than one critical weakness and should not be relied upon to provide an accurate and comprehensive summary of the available studies”.

* Authors provide 7 suggested “critical” items in the AMSTAR 2 tool. Other items are considered “non-critical” weaknesses.

If systematic reviews report GRADE (grading of recommendations, assessment development and evaluation) assessments of outcome certainty, these will be extracted. No new GRADE assessments will be conducted, as no additional meta-analysis will be performed.

Original authors will not be contacted for results and no further statistical analysis will be performed on the data, in line with JBI methodology. ^28^ Alternative umbrella review methodologies suggest re-analysis of the original systematic review data. ^33^ After consideration of this approach, we believe this is not a suitable method in this study for two reasons ^1^: heterogeneous studies would render this methodology technically challenging and potentially inappropriate, ^2^ repeating the analysis of already published studies is not the objective of this review, rather we aim to critically evaluate the available data, which may inform improved future systematic reviews and observational research.

### Presentation of results

We will use the PRISMA 2020 reporting checklist for systematic reviews, in the absence of a corresponding document for umbrella reviews. A narrative description and interpretation of the findings will be given alongside each table. Summary figures may be used to assist interpretation of the data.

### Patient and public involvement

We will discuss the result with people with lived experience of liver disease, including peer support workers living with CHB.

### Ethics and dissemination

The results will be published in a PhD thesis, submitted for consideration for oral and/or poster presentations at international conferences and for peer review and publication in scientific journals. No ethical approval was required for this study, which is using already published systematic reviews and meta-analyses.

## Discussion

We describe a protocol for an umbrella review to critically appraise and systematically summarise data from systematic reviews and meta-analyses investigating the effect of MASLD on liver and viral outcomes in people living with CHB.

The main strength of this review is that it tackles an area of inconsistency which is presenting a current challenge to clinical and public health practice. ^34^ The current literature creates uncertainties for clinicians making management decisions and development of relevant public health approaches, therefore a systematic summary and critical evaluation of published systematic reviews and meta-analyses is an important unmet need. Umbrella review methodology provides a framework to summarise published systematic reviews and meta-analyses, which will help us to examine the underlying reasons for conflicting results, and to identify and guide future research priorities.

A limitation of this study will be the heterogeneity of the literature, which is a reflection of the substantial diversity of populations and disease characteristics of people living with CHB and MASLD. We have taken the approach of maintaining broad inclusion criteria, to capture study characteristics which may drive divergent results. However, diverse populations even within studies, for example differences in antiviral treatment status, participant countries of origin and methods of SLD diagnosis may be difficult to record, and depend on the detail of reporting by authors. These nuances in the data may lead to challenges in interpretation of results. However, this heterogeneity is representative of the current literature, and we will endeavour to report our findings as completely and systematically as possible, acknowledging gaps where necessary.

Finally, we are only including English language studies in this umbrella review. While this pragmatic approach may miss some data, we are reasonably confident that the large majority of CHB/MASLD data is published in English. We will report if any studies were excluded due to language.

This umbrella review will provide a much-needed systematic collation of the evidence for disease outcomes in people living with CHB and MASLD, aiding clinician decision making, highlighting important research gaps and evidence that supports global targets to reduce liver cancer mortality and elimination of viral hepatitis as a public health threat.

## Data Availability

Data will be published along with the manuscript, either in supplementary material or in an online repository, such as Figshare.

## Data statement

No data associated with this article.

### Extended data

Figshare. What is the impact of metabolic dysfunction-associated steatotic liver disease (MASLD) on outcomes in people living with chronic hepatitis B (CHB)? An umbrella review protocol - PRISMA 2020 checklist. https://doi.org/10.6084/m9.figshare.31981305. ^35^

The AMSTAR-2 excel tool is also available online (adapted by the original article). ^32^ https://doi.org/10.6084/m9.figshare.31981317. ^36^

Data is available under the terms of the Creative Commons Attribution 4.0 International license.
