# Supplementary material for: What is the impact of metabolic dysfunction-associated steatotic liver disease (MASLD) on outcomes in people living with chronic hepatitis B (CHB)? An umbrella review protocol
Source: Wellcome Open Res. 2026 Aug 13;11:285. Originally published 2026 May 14. [Version 2] doi: 10.12688/wellcomeopenres.26466.2 (PMC13562955; doi:10.12688/wellcomeopenres.26466.2)
Supplement: Peer review history

# Reviewer report: Reviewer response for version 2

**DOI:** 10.21956/wellcomeopenres.30160.r164200

**Published:** 2026-09-10

**Authors:** Golo Ahlenstiel

**Affiliations:** 1 Western Sydney University, Sydney, Australia

All my queries have been addressed

Is the study design appropriate for the research question?

Yes

Is the rationale for, and objectives of, the study clearly described?

Yes

Are sufficient details of the methods provided to allow replication by others?

Yes

Are the datasets clearly presented in a useable and accessible format?

Not applicable

Reviewer Expertise:

Gastroenterology, Hepatology, Immunology,, viral hepatitis

I confirm that I have read this submission and believe that I have an appropriate level of expertise to confirm that it is of an acceptable scientific standard.

# Reviewer report: Reviewer response for version 1

**DOI:** 10.21956/wellcomeopenres.29145.r157193

**Published:** 2026-06-22

**Authors:** Golo Ahlenstiel

**Affiliations:** 1 Western Sydney University, Sydney, Australia

This manuscript appears to list the intention of undertaking a research study.

 The methods are well described. It appears to be a meta-analysis.

 While I appreciate that this is a research protocol, I am unsure what the originality or novelty is in this manuscript.

 The purpose for indexing this should be clarified.

Is the study design appropriate for the research question?

Yes

Is the rationale for, and objectives of, the study clearly described?

Yes

Are sufficient details of the methods provided to allow replication by others?

Yes

Are the datasets clearly presented in a useable and accessible format?

Not applicable

Reviewer Expertise:

Gastroenterology, Hepatology, Immunology,, viral hepatitis

I confirm that I have read this submission and believe that I have an appropriate level of expertise to confirm that it is of an acceptable scientific standard, however I have significant reservations, as outlined above.

# Response

**Published:** 2026-07-26

**Authors:** Emily Martyn

**Affiliations:** Matthews Laboratory, The Francis Crick Institute, London, England, UK

**Author response: **Thank you to the author for taking the time to review this study. This manuscript appears to list the intention of undertaking a research study.

**Author response: **This manuscript is submitted in the category of a research protocol, which is a standard approach to optimise transparency and reproducibility, to minimise bias by ensuring that aims and analytical approaches are fully developed before the data collection and analysis is started. Pre-publishing a protocol also provides the benefit of external peer review, which adds expert objective oversight and supports optimisation of the protocol.   The methods are well described. It appears to be a meta-analysis.

**Author response: **Our paper is not describing the intention of undertaking a meta-analysis but sets out (as already clearly stated in the title, abstract and methods) to undertake an Umbrella Review. This is a standardised methodological approach to assimilating data from existing systematic reviews and meta-analyses.   While I appreciate that this is a research protocol, I am unsure what the originality or novelty is in this manuscript.

**Author response: **The originality and novelty is already stated clearly in the abstract and introduction, e.g.“Current literature, including over ten systematic reviews and meta-analyses, yields conflicting evidence…”. We have explained that the intersection of CHB and MASLD has not been consistently described or analysed, despite the very substantial threats to global health posed by each of these conditions.   The purpose for indexing this should be clarified.

**Author response: **The paper should be indexed for the reasons set out above - this supports good research integrity and accountability in the research process. By making study objectives, methods, outcomes, and planned analyses publicly available in advance, we create a permanent record against which the final study can be assessed. This helps reduce the risks of selective outcome reporting, post hoc hypothesis generation, and data-driven analytical decisions, thereby strengthening the credibility and reproducibility of research findings. Protocol publication also enables external scrutiny of the study design, facilitates collaboration, reduces unnecessary duplication of research effort, and demonstrates adherence to recognised standards of responsible research conduct. These principles are reflected in international guidance, including the SPIRIT (Standard Protocol Items: Recommendations for Interventional Trials) guidelines, recommendations of the International Committee of Medical Journal Editors (ICMJE), and policies of major research funders and regulators.

# Reviewer report: Reviewer response for version 1

**DOI:** 10.21956/wellcomeopenres.29145.r156239

**Published:** 2026-06-22

**Authors:** Hans Tillmann

**Affiliations:** 1 East Carolina University, Greenville, North Carolina, USA

**Your review: **This is report on a proposal for an umbrella review for the role of MASLD on outcome in patients with hepatitis B.

 The authors / study team failed in the current version to outline the controversies; this should be outlined. Is it related to better or worse liver disease, is it related to overall vs. liver related mortality? Depending on the controversies different approaches to address them may be needed.

 Given this crucial but easy to overcome shortcomings, I recommend revising the manuscript for a protocol prior to acceptance.

 Another concern is that in my understanding of their proposal, the authors seem to rule out being willing to run their own meta-analysis, which may however, potentially be important to explore of controversies may have arisen from mistakes in prior meta-analysis, which may not be discoverable without running their own analysis? If result differ due to included or excluded studies, a run of a meta-analysis to overcome this controversy may be required.

 However, an umbrella review can only be as good as the underlying data, where re-evaluating underlying data may be required, if suspicion arises, that conflicting assessments arose from methodological flaws.

 As most people with MASLD do not liver from liver related complication but cardiovascular disease or cancer, such analysis should be included too.

 Table 1 list “HBsAg seroclearance (loss of detectable surface antigen in the blood) in people living with CHB not on antiviral treatment.” However, it may be worth stratifying by antiviral therapy status, vs. only looking at treatment naïve patients, as steatosis may also modify HBsAg loss on antiviral therapy too.

 Another concern I would see as a potential obstacle, is that a use of meta-analysis may not be able to dissect MASLD defined by presence of diabetes vs. MASLD defined by presence of hypertension.

 Minor point:

 Table 3 lists HBeAg loss/ sero-conversion, but for HBsAg only HBsAg loss, not sero-conversion, was this an oversight?

Is the study design appropriate for the research question?

Partly

Is the rationale for, and objectives of, the study clearly described?

Partly

Are sufficient details of the methods provided to allow replication by others?

Partly

Are the datasets clearly presented in a useable and accessible format?

Partly

Reviewer Expertise:

NA

I confirm that I have read this submission and believe that I have an appropriate level of expertise to confirm that it is of an acceptable scientific standard, however I have significant reservations, as outlined above.

# Response

**Published:** 2026-07-26

**Authors:** Emily Martyn

**Affiliations:** Matthews Laboratory, The Francis Crick Institute, London, England, UK

**Response to** **Reviewer 2:**

**Author response: **We thank the reviewer for taking the time to read and provide feedback on the manuscript **.**

 Your review: This is report on a proposal for an umbrella review for the role of MASLD on outcome in patients with hepatitis B. The authors / study team failed in the current version to outline the controversies; this should be outlined.

**Author response: **We have added to the introduction to add further clarity, as follows: ‘We have previously undertaken an assessment of the literature (in a narrative review format (Martyn et al. 2026)) finding conflict in conclusions drawn from meta-analyses. For example, some studies report an increased risk of HCC associated with MASLD and CHB (compared to CHB alone) (Mao et al. 2023; Zhou et al. 2023; Han et al. 2023) and others report a lower risk (Wong et al. 2023). Similarly, responses to antiviral therapy for HBV among people living with MASLD are reported to be less robust in some studies (Jiang et al. 2021; Liu et al. 2024; Zeng et al. 2024), but other analyses do not find this effect (Rui et al. 2024; Zhou et al. 2023).’   We would also like to point the reviewer to the following existing text which further highlights the controversies:

- Abstract: “Current literature, including over ten systematic reviews and meta-analyses, yields conflicting evidence about the interplay between CHB and MASLD”.
- Introduction: we explain the current field, reporting that ‘the conclusions drawn by these [existing] reviews are conflicting, and the underlying reasons for inconsistencies are not fully understood’.
- Discussion: we comment on “heterogeneity of the literature, which is a reflection of the substantial diversity of populations and disease characteristics of people living with CHB and MASLD”.

  Is it related to better or worse liver disease, is it related to overall vs. liver related mortality?  

**Author response: **In the introduction section, we clearly state: “Our objective is to collate, systematically and critically evaluate, and identify gaps in the data for the effect of MASLD on outcomes of HBV infection, including HBsAg clearance (functional cure), response to antiviral therapy, and rates of liver inflammation, fibrosis, cirrhosis and hepatocellular carcinoma in people living with CHB”. In response to the author’s comment we have added all-cause mortality to the primary outcomes. We are agnostic to ‘better’ or ‘worse’ outcomes, as this will be part of the findings of the final umbrella review. The outcome data that will be collected from each study and collated for analysis are clearly set out in Table 3.   We do introduce the topic with a clear narrative which states that the reason why this topic is important is (a) because there are international targets to reduce mortality from viral hepatitis, and (b) because hepatocellular carcinoma mortality rates continue to rise globally, with MASLD and CHB remaining leading causes.

   Depending on the controversies different approaches to address them may be needed.  

**Author response: **The primary outcome for this protocol (and the research it describes) is to take a retrospective approach to describing and collating the existing evidence using a meticulous, evidence-based approach. We agree that in future, for new prospective studies, different approaches may be needed - this is potentially a recommendation that will arise as part of the discussion of the final research paper but is not a component of the protocol that is presented here.  

 Given this crucial but easy to overcome shortcomings, I recommend revising the manuscript for a protocol prior to acceptance.  

**Author response: **We are not certain in what way the reviewer intends the manuscript to be revised for a protocol - as it already is a protocol. However, we point to several specific modifications undertaken in response to feedback from reviewer 1, which have strengthened the methodology and analytic frameworks we intend to apply. We hope these updates also address any additional concerns from reviewer 2. We also refer to feedback from reviewer 1, who states ‘the protocol is well written, addresses an important clinical question, and follows an established umbrella review framework’.

   Another concern is that in my understanding of their proposal, the authors seem to rule out being willing to run their own meta-analysis, which may however, potentially be important to explore of controversies may have arisen from mistakes in prior meta-analysis, which may not be discoverable without running their own analysis? If result differ due to included or excluded studies, a run of a meta-analysis to overcome this controversy may be required. However, an umbrella review can only be as good as the underlying data, where re-evaluating underlying data may be required, if suspicion arises, that conflicting assessments arose from methodological flaws.  

**Author response: **At the outset of this project, we considered undertaking a further meta-analysis. However, we were unable to justify this approach given that over ten other such studies have already been conducted (as we explain in the abstract and introduction). On these grounds, a more considered approach is to take a systematic approach to reviewing the existing landscape, such that we can identify and report on the reasons for different outcomes, heterogeneity, and controversies. In the absence of this understanding, it would be difficult to design and conduct an improved study, and we would simply be duplicating efforts that have already been published by other teams.   The reviewer refers to ‘mistakes’ in prior meta-analysis - we believe there is a wider nuance, which is addressed by our proposed study, which is that we take a granular view to thoroughly understanding the quality, transparency, and methodological approaches taken by previous studies. This will allow us to make recommendations for future studies to improve the quality of new primary data, and any subsequent meta-analysis. We agree that any review is ‘only as good as the underlying data’, and this point will be addressed in our quality appraisal which will take a systematic approach to identifying flaws, weaknesses or inconsistencies in the studies which we include.  

 As most people with MASLD do not liver from liver related complication but cardiovascular disease or cancer, such analysis should be included too.  

**Author response: **There appears to be an error in this comment - is the reviewer referring to mortality from liver related complications? We clearly set out an a priori objective which seeks to determine the relationship between CHB and MASLD. Cardiovascular disease could be the topic of an entirely separate review protocol, but is not within scope of the study we have set out to do here. Liver cancer is clearly included, and is one of the outcomes considered by the studies that will be captured by the umbrella review (see table 1 / “outcomes” section)  

 Table 1 list “HBsAg seroclearance (loss of detectable surface antigen in the blood) in people living with CHB not on antiviral treatment.” However, it may be worth stratifying by antiviral therapy status, vs. only looking at treatment naïve patients, as steatosis may also modify HBsAg loss on antiviral therapy too.  

**Author response:**  In our review we already intend to look at surface antigen seroclearance +/- seroconversion in both the context of response to antiviral treatment, and without antiviral treatment. We will be guided by what is reported in the published systematic reviews as to whether we can provide granular detail on whether the participants are treatment naive, but intend to report this if possible. We direct the reviewer to two different bullet points in the table 1 “outcome” row:  

- “HBsAg seroclearance (loss of detectable surface antigen in the blood) in people living with CHB not on antiviral treatment.
-  Response to antiviral treatment (NAs and/or interferon)
  - Biochemical (e.g. ALT normalisation) or viral (e.g. HBeAg or HBsAg seroconversion, viral load suppression)”

  Another concern I would see as a potential obstacle, is that a use of meta-analysis may not be able to dissect MASLD defined by presence of diabetes vs. MASLD defined by presence of hypertension.

**Author response: **As MASLD has been defined by an international Delphi process and is now accepted consensus in the field, it is appropriate for us to stick as closely as possible to this terminology and definition. MASLD includes hepatic steatosis plus at least one of overweight/obesity, hypertension, dyslipidaemia or diabetes. Some individuals will have only one of these criteria, and others may have many. In most existing studies, the presence of these individual factors is not reported as disaggregated data, and we therefore need to stick to using the umbrella term of MASLD. Including future aspirations for individual studies that more carefully consider individual risk factors  

 Minor point: Table 3 lists HBeAg loss/ sero-conversion, but for HBsAg only HBsAg loss, not sero-conversion, was this an oversight?

**Author response**: This was an oversight and we have now added loss and seroconversion, into the “outcomes” row in table 1 and the new “outcomes” section in the text. See the amended  bullet point below from table 1:   “HBsAg seroclearance (loss of detectable HBsAg in the blood) and/ or seroconversion (loss of HBsAg and appearance of anti- hepatitis B surface antibodies) in people living with CHB not on antiviral treatment.”  

**References (also updated in the revised manuscript)** Han, Cheng-Long, Bao-Wen Tian, Chun-Cheng Yang, et al. 2023. “The Association of Fatty Liver and Risk of Hepatocellular Carcinoma in HBV or HCV Infected Individuals: A Systematic Review and Meta-Analysis.” *Expert Review of Gastroenterology & Hepatology* 17 (2): 189–198. Jiang, Daixi, Can Chen, Xiaoxiao Liu, et al. 2021. “Concurrence and Impact of Hepatic Steatosis on Chronic Hepatitis B Patients: A Systematic Review and Meta-Analysis.” *Annals of Translational Medicine* 9 (23): 1718. Liu, Shi-Yi, Dian Wang, Jing Liu, Lu-Ping Yang, and Gong-Ying Chen. 2024. “Influence of Nonalcoholic Fatty Liver Disease on Response to Antiviral Treatment in Patients with Chronic Hepatitis B: A Meta-Analysis.” *World Journal of Hepatology* 16 (3): 465–476. Mao, Xianhua, Ka Shing Cheung, Chengzhi Peng, et al. 2023. “Steatosis, HBV-Related HCC, Cirrhosis, and HBsAg Seroclearance: A Systematic Review and Meta-Analysis.” *Hepatology (Baltimore, Md.)* 77 (5): 1735–1745. Martyn, Emily, Alejandro Arenas-Pinto, Richard Gilson, et al. 2026. “Diagnosing and Defining MASLD in People Living with Chronic Hepatitis B.” *Communications Medicine* 6 (1). https://doi.org/10.1038/s43856-026-01383-2. Rui, Fajuan, Elizabeth Garcia, Xinyu Hu, et al. 2024. “Antiviral Therapy Response in Patients with Chronic Hepatitis B and Fatty Liver: A Systematic Review and Meta-Analysis.” *Journal of Viral Hepatitis* 31 (7): 372–382. Wong, Yu Jun, Vy H. Nguyen, Hwai-I Yang, et al. 2023. “Impact of Fatty Liver on Long-Term Outcomes in Chronic Hepatitis B: A Systematic Review and Matched Analysis of Individual Patient Data Meta-Analysis.” *Clinical and Molecular Hepatology* 29 (3): 705–720. Zeng, Georgia, Benjamin R. Holmes, Saleh A. Alqahtani, Upkar S. Gill, and Patrick T. F. Kennedy. 2024. “The Co-Existence of NAFLD and CHB Is Associated with Suboptimal Viral and Biochemical Response to CHB Antiviral Therapy: A Systematic Review and Meta-Analysis.” *Frontiers in Gastroenterology (Lausanne, Switzerland)* 3 (January): 1333988. Zhou, Run, Luping Yang, Binbin Zhang, et al. 2023. “Clinical Impact of Hepatic Steatosis on Chronic Hepatitis B Patients in Asia: A Systematic Review and Meta-Analysis.” *Journal of Viral Hepatitis* 30 (10): 793–802.

# Reviewer report: Reviewer response for version 1

**DOI:** 10.21956/wellcomeopenres.29145.r155603

**Published:** 2026-06-12

**Authors:** Yoscelina E. Martinez-Lopez

**Affiliations:** 1 University of Texas Health Science Center at Houston, Brownsville, TX, USA

This manuscript presents a protocol for an umbrella review aiming to evaluate the impact of metabolic dysfunction-associated steatotic liver disease (MASLD) on liver-related and virological outcomes among people living with chronic hepatitis B (CHB). The authors propose to systematically identify, appraise, and synthesize evidence from published systematic reviews and meta-analyses using Joanna Briggs Institute (JBI) umbrella review methodology and AMSTAR-2 quality assessment. The topic is highly relevant given the increasing prevalence of MASLD worldwide, the substantial global burden of CHB, and the growing number of systematic reviews that have reported conflicting findings regarding the interaction between these two conditions. Overall, the protocol is well written, addresses an important clinical question, and follows an established umbrella review framework.

 Major Comments:

 1. The most important methodological limitation of this protocol is the absence of a strategy to assess overlap among included systematic reviews and meta-analyses. Because the authors intend to include multiple reviews addressing similar research questions, there is a substantial risk that the same primary observational studies will be represented repeatedly across reviews. The protocol does not describe how overlap among primary studies will be quantified or managed. This omission is important because overlap can artificially inflate the perceived strength of evidence and may influence conclusions about the consistency of findings. The authors should consider constructing a citation matrix and calculating the Corrected Covered Area (CCA), which is widely recommended in umbrella review methodology. Furthermore, they should explain how highly overlapping reviews will be handled and whether certain reviews will be prioritized when substantial redundancy exists.

 2. The principal rationale for conducting this umbrella review is the existence of conflicting evidence regarding the impact of MASLD on outcomes among people living with chronic hepatitis B. However, the protocol does not specify how conflicting conclusions will be interpreted or synthesized. It would be helpful for the authors to clarify whether greater weight will be assigned to reviews with higher methodological quality, larger sample sizes, more recent search periods, or more comprehensive inclusion criteria.

 3. The discussion regarding the certainty of evidence also requires further development. The authors state that existing GRADE assessments will be extracted when available, but that no new certainty assessments will be conducted. While this approach is understandable within the context of an umbrella review, it raises concerns regarding consistency. Many systematic reviews may not have conducted GRADE assessments, and those that did may have used different approaches. The authors should provide a stronger justification for this decision and explain how confidence in the body of evidence will ultimately be communicated.

 4. The restriction to English-language publications introduces the possibility of language bias. This issue is particularly relevant in hepatitis B research, where a big proportion of the literature originates from East Asian countries such as China, Taiwan, Korea, and Japan. Although the authors acknowledge this limitation, the discussion remains relatively brief and does not adequately address the potential impact of excluding non-English reviews. A more detailed justification for this restriction, together with a discussion of its implications for the comprehensiveness of the evidence base, would strengthen the protocol.

 5. The protocol does not address the potential contribution of grey literature. The authors should explicitly justify this decision and discuss its potential implications.

 6. The search strategy itself may benefit from further refinement. Although the selected databases are appropriate and likely to capture much of the relevant literature, the search terms appear largely keyword-based and relatively simple. The protocol does not indicate whether controlled vocabulary terms such as MeSH or EMTREE headings will be used. Given the importance of comprehensive evidence identification in umbrella reviews, the authors should consider describing the use of controlled vocabulary and reporting whether the search strategy underwent peer review, for example, through the PRESS framework.

 7. Another challenge concerns the inclusion of multiple related but distinct definitions of steatotic liver disease, including MASLD, MAFLD, NAFLD, SLD, and hepatic steatosis. Although the authors appropriately acknowledge the evolution of nomenclature, these entities are not fully interchangeable and may identify somewhat different patient populations. The protocol would benefit from a more detailed explanation of how these varying definitions will be handled analytically. At present, it remains unclear whether differences in terminology will be explored as a potential source of heterogeneity or whether subgroup analyses will be conducted according to disease definition.

 8. The protocol repeatedly highlights heterogeneity as a major challenge within the literature. However, there is no formal framework for evaluating potential sources of heterogeneity. Important factors that may contribute to divergent findings include antiviral treatment status, geographic region, HBV genotype, method of steatosis diagnosis, study design, and the specific MASLD/MAFLD/NAFLD definitions employed. A structured approach for identifying and reporting these sources of heterogeneity would strengthen the review.

 9. Although AMSTAR-2 is an appropriate and widely accepted tool for assessing the methodological quality of systematic reviews, it evaluates review conduct rather than the certainty of evidence generated by the underlying primary studies. The protocol would benefit from additional discussion regarding how AMSTAR-2 findings will influence the interpretation of the evidence.

 Minor Comments

 1. The introduction is generally well written and provides appropriate context for the proposed review. However, some sections occasionally read as advocacy rather than objective scientific justification. Statements regarding the urgency of the topic and the implications of recent WHO guideline changes should be presented in a more balanced manner. They should clearly distinguish established evidence from interpretation.

 2. The objectives are comprehensive but encompass a wide range of both virological and liver-related outcomes. The manuscript would benefit from clearly distinguishing primary from secondary outcomes, as this would provide greater clarity on the review's main focus.

 3. Although the discussion appropriately acknowledges heterogeneity and language restrictions, it would benefit from a broader consideration of overlap bias, review redundancy, certainty of evidence, and publication bias, as these are important methodological challenges in umbrella reviews.

Is the study design appropriate for the research question?

Partly

Is the rationale for, and objectives of, the study clearly described?

Yes

Are sufficient details of the methods provided to allow replication by others?

Partly

Are the datasets clearly presented in a useable and accessible format?

Not applicable

Reviewer Expertise:

Infectious disease epidemiology, tuberculosis, chronic disease comorbidities, systematic reviews, meta-analysis and evidence synthesis, observational study methodology, biostatistics, and global health research.

I confirm that I have read this submission and believe that I have an appropriate level of expertise to confirm that it is of an acceptable scientific standard, however I have significant reservations, as outlined above.

# Response

**Published:** 2026-07-26

**Authors:** Emily Martyn

**Affiliations:** Matthews Laboratory, The Francis Crick Institute, London, England, UK

**Reviewer 1** This manuscript presents a protocol for an umbrella review aiming to evaluate the impact of metabolic dysfunction-associated steatotic liver disease (MASLD) on liver-related and virological outcomes among people living with chronic hepatitis B (CHB). The authors propose to systematically identify, appraise, and synthesize evidence from published systematic reviews and meta-analyses using Joanna Briggs Institute (JBI) umbrella review methodology and AMSTAR-2 quality assessment. The topic is highly relevant given the increasing prevalence of MASLD worldwide, the substantial global burden of CHB, and the growing number of systematic reviews that have reported conflicting findings regarding the interaction between these two conditions. Overall, the protocol is well written, addresses an important clinical question, and follows an established umbrella review framework.

**Authors’ comment:** Thank you to the reviewer for helpful and positive feedback.  

**Major Comments:**

 1. The most important methodological limitation of this protocol is the absence of a strategy to assess overlap among included systematic reviews and meta-analyses. Because the authors intend to include multiple reviews addressing similar research questions, there is a substantial risk that the same primary observational studies will be represented repeatedly across reviews. The protocol does not describe how overlap among primary studies will be quantified or managed. This omission is important because overlap can artificially inflate the perceived strength of evidence and may influence conclusions about the consistency of findings. The authors should consider constructing a citation matrix and calculating the Corrected Covered Area (CCA), which is widely recommended in umbrella review methodology. Furthermore, they should explain how highly overlapping reviews will be handled and whether certain reviews will be prioritized when substantial redundancy exists.

**Authors’ response:** Thank you for the comment. We agree a citation matrix would be a useful addition to this study and will also calculate the CCA to quantify the overlap between studies. We have added the following paragraph to the “Analysis of Evidence” section: “For each primary outcome (table 1) we will generate a citation matrix to visualise the overlap of primary studies across systematic reviews, where the columns are the systematic reviews and the rows are the primary studies. A heatmap will be used to visualise the overlap between studies. To quantify the degree of primary study overlap between reviews, we will  calculate the Corrected Covered Area (CCA) using the following formula (Pieper et al. 2014):  CCA = N - r / rc - r *Where N is the number of included publications in evidence syntheses, r is the number of indexed publications (rows), c is the number of reviews (columns). * A guide to interpretation of the CCA, as per Pieper’s original paper, is in ( **Table 5**)”

 2. The principal rationale for conducting this umbrella review is the existence of conflicting evidence regarding the impact of MASLD on outcomes among people living with chronic hepatitis B. However, the protocol does not specify how conflicting conclusions will be interpreted or synthesized. It would be helpful for the authors to clarify whether greater weight will be assigned to reviews with higher methodological quality, larger sample sizes, more recent search periods, or more comprehensive inclusion criteria.

**Author response: **Points 2 and 3 are related, therefore we will address them together; please see below specific response to point 3.

 3. The discussion regarding the certainty of evidence also requires further development. The authors state that existing GRADE assessments will be extracted when available, but that no new certainty assessments will be conducted. While this approach is understandable within the context of an umbrella review, it raises concerns regarding consistency. Many systematic reviews may not have conducted GRADE assessments, and those that did may have used different approaches. The authors should provide a stronger justification for this decision and explain how confidence in the body of evidence will ultimately be communicated.

**Author response: **Points 2 and 3 are related, therefore we will address them together. In response to this point, and to maintain consistency, we have opted to use the criteria suggested by Belbasis and colleagues to rate the confidence of each review outcome (see citations in the next paragraph). These criteria are based on number of cases, effect p value, heterogeneity, 95% prediction value and other tests of small study effects and excess significance bias. We have added the following paragraph to the “Analysis of evidence” section: “We will use criteria suggested by Belbasis and colleagues to rate our confidence in each individual systematic review outcome (Table 6) (Bellou et al. 2017; Belbasis et al. 2015). There is a precedent for using this confidence rating system in previously published umbrella reviews, and they are designed for  observational associations (Fusar-Poli and Radua 2018). Based on the review methodological quality and confidence rating for each outcome, we will give an overall confidence rating for each outcome, which will be a judgement by authors EM and JC (with additional input from a third author if conflict cannot be resolved). A narrative justification for the final decision will be included in the manuscript text.”

 4. The restriction to English-language publications introduces the possibility of language bias. This issue is particularly relevant in hepatitis B research, where a big proportion of the literature originates from East Asian countries such as China, Taiwan, Korea, and Japan. Although the authors acknowledge this limitation, the discussion remains relatively brief and does not adequately address the potential impact of excluding non-English reviews. A more detailed justification for this restriction, together with a discussion of its implications for the comprehensiveness of the evidence base, would strengthen the protocol.

**Author response:** We have expanded on our argument for the inclusion of only English language studies, by including this paragraph in the discussion: “For pragmatic reasons, we are only including English language studies in this umbrella review. We hypothesize that the reviews with the highest global impact will be published in English and indexed in one of our selected search databases, and therefore this search strategy will identify the most relevant results. However, we recognize that undertaking a language-restricted approach, and by not searching non-medline indexed Chinese databases e.g. China National Knowledge Infrastructure or Wanfang data, we could increase the chances of missing a potentially relevant review, given the focus on hepatitis B research in East Asian countries such as China, Taiwan and Korea. We will mitigate the risk of bias by reporting if  any studies were excluded due to language and discussing the potential impact of any study omissions on our umbrella review conclusions.”  

 5. The protocol does not address the potential contribution of grey literature. The authors should explicitly justify this decision and discuss its potential implications.

**Author response: **Although we did specify “conference abstracts” in the exclusion criteria, we did not further elaborate on grey literature. In response to the reviewer, we have added “pre-prints” and “theses” to the exclusion criteria (Table 1), and included the following paragraph in the discussion: “In this umbrella review, we opted not to specifically search for or include grey literature (e.g., conference abstracts, preprints, theses, or trial and review registries such as PROSPERO). Given that our topic is already served by multiple peer-reviewed, published systematic reviews and meta-analyses, and given the substantial methodological heterogeneity already present within this peer-reviewed literature, we judged that incorporating non-peer-reviewed sources of variable and unverifiable methodological quality risked introducing additional bias into our credibility assessment, rather than mitigating it.” We have also stated we will not be searching non-English language, non-MEDLINE indexed databases e.g. CKNI.  Please see our response to point 4.

 6. The search strategy itself may benefit from further refinement. Although the selected databases are appropriate and likely to capture much of the relevant literature, the search terms appear largely keyword-based and relatively simple. The protocol does not indicate whether controlled vocabulary terms such as MeSH or EMTREE headings will be used. Given the importance of comprehensive evidence identification in umbrella reviews, the authors should consider describing the use of controlled vocabulary and reporting whether the search strategy underwent peer review, for example, through the PRESS framework.

**Author response:** In response to this reviewer's suggestion, we revisited our search strategy with our institute librarian. We have now done a primary search and an updated search strategy using MeSH and EMTREE headings and no additional studies were identified, so we are confident that our initial search strategy was comprehensive. We are unable  to undertake a full formal PRESS (Peer Review of Electronic Search Strategies) review within the review timeline. However, given the updated search strategy yielded no additional results, we believe this represents a pragmatic quality check given the constraints of the review timeline. In the “Search strategy” section, we have added the following sentence: “We will run an updated search strategy using MeSH and EMTREE headings to determine whether any additional studies are identified, to ensure that our initial search strategy is comprehensive.”

 7. Another challenge concerns the inclusion of multiple related but distinct definitions of steatotic liver disease, including MASLD, MAFLD, NAFLD, SLD, and hepatic steatosis. Although the authors appropriately acknowledge the evolution of nomenclature, these entities are not fully interchangeable and may identify somewhat different patient populations. The protocol would benefit from a more detailed explanation of how these varying definitions will be handled analytically. At present, it remains unclear whether differences in terminology will be explored as a potential source of heterogeneity or whether subgroup analyses will be conducted according to disease definition.

**Author response: **We would like to highlight our table of definitions of SLD which will help the reader to understand the differences between MAFLD, MASLD and NASH. We will document the definition of SLD used by each study. We will be describing the evidence for each outcome from each study, and we will not be combining them. We will narratively describe how the different definitions of SLD used may impact the results. To clarify, we have added a sentence to address this point under “Presentation of results”: “A narrative description and interpretation of the findings will be given alongside each figure and table, and we will discuss the impact of heterogeneity, including different SLD definitions on the our interpretation of the results.” Please see the answer to question 8 for further discussion on this point.

 8. The protocol repeatedly highlights heterogeneity as a major challenge within the literature. However, there is no formal framework for evaluating potential sources of heterogeneity. Important factors that may contribute to divergent findings include antiviral treatment status, geographic region, HBV genotype, method of steatosis diagnosis, study design, and the specific MASLD/MAFLD/NAFLD definitions employed. A structured approach for identifying and reporting these sources of heterogeneity would strengthen the review.

**Author response:  **We will address points 7 & 8 together, as they both refer to the major issue of heterogeneity in the literature. As part of the data collection process, for each systematic review we will extract study characteristics which contribute to heterogeneity in the literature, including definition of steatotic liver disease, method of steatosis diagnosis, study design, geographical restrictions, e-Antigen status, antiviral treatment status and basic population demographics. We recognise HBV genotype as an important and understudied potential source of heterogeneity when considering clinical outcomes in CHB, but this is not a commonly reported variable in the MASLD/CHB field and so we have not incorporated this into our data collection tool.  Our data collection strategy was already detailed in table 3, but we have added this sentence in the “data extraction” section to clarify our approach for our readers: “Data extraction will include capturing key sources of heterogeneity, including the definition of SLD used in the systematic review (e.g. MASLD, NAFLD, hepatic steatosis), method of SLD diagnosis, geographical restrictions, e-Antigen status antiviral treatment status, among others detailed in **table 3**.” We have also added the following section in the “presentation of results” section to highlight how we will present these potential sources of heterogeneity across reviews: “To enable a qualitative comparison of studies and highlight any obvious heterogeneity, we will use a table to describe the PICO characteristics of each included systematic review, including: ·       key exclusion and inclusion criteria (e.g. geographical restriction), ·       overall population demographics (where reported), ·       viral parameters (e.g. number on AVT, or eAg positive), ·       SLD definition and method of SLD diagnosis.” 9. Although AMSTAR-2 is an appropriate and widely accepted tool for assessing the methodological quality of systematic reviews, it evaluates review conduct rather than the certainty of evidence generated by the underlying primary studies. The protocol would benefit from additional discussion regarding how AMSTAR-2 findings will influence the interpretation of the evidence.

**Author response:** To clarify how we will use the AMSTAR-2 findings, we have added the following paragraph into the “presentation of results” section: “For each umbrella review primary outcome, (e.g. HCC risk) we will generate a table with the following headings: ·       review (author, date), ·  outcome definition (in that specific review e.g. HBeAg seroclearance vs seroconversion) ·       effect metric (e.g. odds ratio, hazard ratio), ·       summary effect result and 95% confidence interval, ·       summary effect p value, · *I ^2^* statistic to describe heterogeneity, ·       direction of effect (e.g. decreased/ increased risk of HCC), ·        methodological assessment (AMSTAR-2 overall rating), ·       confidence rating (Belbasis et al., 2015). Based on the evidence provided by each contributing systematic review, we will assign an overall confidence rating for each primary outcome of the umbrella review. The overall rating will be discussed and agreed by two authors (EM and JC) and a third author will be consulted if a consensus cannot be reached.”

 Minor Comments

 1. The introduction is generally well written and provides appropriate context for the proposed review. However, some sections occasionally read as advocacy rather than objective scientific justification. Statements regarding the urgency of the topic and the implications of recent WHO guideline changes should be presented in a more balanced manner. They should clearly distinguish established evidence from interpretation.

**Author response:** We have adjusted the language in the introduction to move the tone away from advocacy towards scientific justification. For example we have changed the original sentence at the end of the first paragraph from: “MASLD prevalence is rising in the context of a global increase in obesity and type 2 diabetes, and a critical convergence of MASLD and CHB is emerging.” to a new sentence: “MASLD prevalence is rising in parallel with increasing rates of obesity and type 2 diabetes, which may lead to an increase in the number of people living with MASLD and CHB.” We have also rewritten the third paragraph, removing phrases such as “we urgently need to better understand the data”, to ensure we are stating facts and scientific justification rather than highlighting a call-to-action. The new paragraph is as follows: “We are not currently on track to achieve World Health Organization (WHO) viral hepatitis elimination targets, which include a 90% reduction in incidence and 65% reduction in mortality from viral hepatitis by 2030 (23,24). Hepatocellular carcinoma mortality rates continue to rise globally, with MASLD and CHB among the major underlying causes (25). In the revised 2024 hepatitis B treatment guidelines, MASLD was included a one factor that may support the intiation of antiviral therapy, although the evidence base informing this recommendation remains limited. (26). At the same time, new pharmacological treatments are becoming available for MASLD, but trials exclude people living with CHB (27). Together, these developments highlight uncertainties regarding the management of individuals living with both MASLD and CHB, and the need to synthesise the available evidence.”

 2. The objectives are comprehensive but encompass a wide range of both virological and liver-related outcomes. The manuscript would benefit from clearly distinguishing primary from secondary outcomes, as this would provide greater clarity on the review's main focus.

**Author response: **In response to the reviewer's suggestion, we have added a new "Outcomes" subheading to clearly distinguish the primary and secondary outcomes of interest. As the overarching objective of the review is already described in the final paragraph of the Introduction, we have removed the original "Objectives" section to avoid duplication. The revised "Outcomes" section is reproduced below. “The following outcomes will be evaluated in people living with both MASLD and CHB, compared to CHB alone: Primary outcomes: 1.     Liver disease progression: risk (e.g. hazard ratio, risk ratio, odds ratio) of liver inflammation (ALT), fibrosis, cirrhosis and HCC 2.     Hepatitis B surface antigen (HBsAg) seroclearance (loss of detectable HBsAg in the blood) and/ or seroconversion (appearance of anti-hepatitis B surface antibodies), not on antiviral treatment 3.     Antiviral treatment response (nucleoside analogues and/or interferon): a.     biochemical – ALT normalisation b.     serological – hepatitis B e-antigen (HBeAg) or HBsAg seroconclearance and/or seroconversion c.     virological – HBV DNA suppression   Secondary outcome (irrespective of antiviral treatment response): ●       HBV viral load e.g. mean difference between MASLD and non-MASLD groups (cross sectional studies), change over time (longitudinal studies).”

 3. Although the discussion appropriately acknowledges heterogeneity and language restrictions, it would benefit from a broader consideration of overlap bias, review redundancy, certainty of evidence, and publication bias, as these are important methodological challenges in umbrella reviews.

**Author response:** We believe we have now adequately addressed these issues throughout the protocol by responding to the reviewer’s major comments. However, in response to this comment, we have elaborated on these methodological issues in the discussion by adding the following paragraphs: “Overlap of primary studies across systematic reviews is an important methodological consideration in umbrella reviews, as it may result in the same evidence contributing disproportionately to the overall synthesis and increase confidence in findings that are supported by relatively few independent studies. As this umbrella review will synthesise and critically appraise existing systematic reviews, rather than conducting additional meta-analyses to generate pooled estimates, the potential impact of overlap on quantitative effect estimates will be reduced. However, we will quantify primary study overlap using citation matrices and the Corrected Covered Area (CCA), allowing the extent of overlap to be considered when interpreting the findings. Furthermore, where reviews with a high degree of study overlap reach contrasting conclusions, examining differences in their methodology may help explain inconsistencies within the literature. Certainty of the evidence and publication bias are additional challenges that influence the interpretation of umbrella reviews. We will assess evidence certainty using predefined criteria, while the methodological quality assessment (AMSTAR 2) includes consideration of whether publication bias has been appropriately evaluated by the included systematic reviews. Together, these assessments will enable us to interpret the review findings in the context of both the strength of the available evidence and potential sources of bias.” **References (also updated in the revised manuscript)** Belbasis, Lazaros, Vanesa Bellou, Evangelos Evangelou, John P. A. Ioannidis, and Ioanna Tzoulaki. 2015. “Environmental Risk Factors and Multiple Sclerosis: An Umbrella Review of Systematic Reviews and Meta-Analyses.” *Lancet Neurology* 14 (3): 263–273. Bellou, Vanesa, Lazaros Belbasis, Ioanna Tzoulaki, Lefkos T. Middleton, John P. A. Ioannidis, and Evangelos Evangelou. 2017. “Systematic Evaluation of the Associations between Environmental Risk Factors and Dementia: An Umbrella Review of Systematic Reviews and Meta-Analyses.” *Alzheimer’s & Dementia: The Journal of the Alzheimer's Association* 13 (4): 406–418. Fusar-Poli, Paolo, and Joaquim Radua. 2018. “Ten Simple Rules for Conducting Umbrella Reviews.” *Evidence-Based Mental Health* 21 (3): 95–100.
